# Feasibility and Compliance of Stool Collection for Future Microbiome-Based Colorectal Cancer Screening: Preliminary Findings from a Prospective Multicenter FIT-Positive Cohort

**DOI:** 10.3390/microorganisms14071564

**Published:** 2026-07-17

**Authors:** Andrea Severino, Debora Rondinella, Simone Varca, Tommaso Schepis, Serena Porcari, Piergiorgio Bisegna, Ernesto Margarita, Federico Barbaro, Silvia Pecere, Rossella Maresca, Daniela Feliciani, Barbara Funaro, Anna Latiano, Orazio Palmieri, Alessandro Azzarone, Paola Cesaro, Daniele Salvi, Carla Treppiccione, Gianmarco Piccinno, Nicola Segata, Cristiano Spada, Antonio Gasbarrini, Gianluca Ianiro

**Affiliations:** 1 Department of Translational Medicine and Surgery, Università Cattolica del Sacro Cuore, 00168 Rome, Italy; andrea.severino@guest.policlinicogemelli.it (A.S.); debora.rondinella@policlinicogemelli.it (D.R.); simone.varca@guest.policlinicogemelli.it (S.V.); tommaso.schepis@guest.policlinicogemelli (T.S.); serena.porcari@policlinicogemelli.it (S.P.); piergiorgio.bisegna01@icatt.it (P.B.); ernesto.margarita00@gmail.com (E.M.); federico.barbaro@policlinicogemelli.it (F.B.); silvia.pecere@policlinicogemelli.it (S.P.); rossella.maresca12@gmail.com (R.M.); daniela.feliciani@policlinicogemelli.it (D.F.); barbara.funaro@policlinicogemelli.it (B.F.); carla.treppiccione@poliambulanza.it (C.T.); cristiano.spada@policlinicogemelli.it (C.S.); antonio.gasbarrini@unicatt.it (A.G.); 2 Department of Medical and Surgical Sciences, UOC CEMAD Centro Malattie dell’Apparato Digerente, Medicina Interna e Gastroenterologia, Fondazione Policlinico Universitario Agostino Gemelli IRCCS, 00168 Rome, Italy; 3 UOC Endoscopia Digestiva Chirurgica, Fondazione Policlinico Universitario Agostino Gemelli IRCCS, 00168 Rome, Italy; 4 Gastrointestinal Disorders Research Unit, Fondazione IRCCS Casa Sollievo della Sofferenza, 71013 San Giovanni Rotondo, Italy; a.latiano@operapadrepio.it (A.L.); o.palmieri@operapadrepio.it (O.P.); 5 Digestive Endoscopy and Screening CCR Unit, Di Venere Hospital, 70131 Bari, Italy; alessandroantonio.azzarone@asl.bari.it; 6 Department of Gastroenterology and Endoscopy, Fondazione Poliambulanza Istituto Ospedaliero, 25124 Brescia, Italy; paola.cesaro@poliambulanza.it (P.C.); daniele.salvi@poliambulanza.it (D.S.); 7 Department of Cellular, Computational and Integrative Biology, University of Trento, 38123 Trento, Italy; gianmarco.piccinno@unitn.it (G.P.); nicola.segata@unitn.it (N.S.)

**Keywords:** colorectal cancer, screening, colonoscopy, gut microbiota, microbial biomarkers

## Abstract

Colorectal cancer (CRC) remains a major global health burden, and early detection through population-based screening programs significantly reduces both incidence and mortality. Although gut microbiome-based biomarkers have emerged as promising non-invasive tools for CRC detection, limited evidence is available regarding patient acceptance and compliance with microbiome-based screening studies, factors that may influence their future implementation in clinical practice. We conducted a preliminary analysis of an ongoing multicenter, prospective observational study designed to develop a gut microbiome-based diagnostic tool for CRC and advanced colorectal adenomas in fecal immunochemical test (FIT)-positive individuals. The primary objective of this preliminary analysis was to evaluate patient acceptance and compliance with participation in a microbiome-based study within an organized CRC screening setting. Secondary objectives included describing the clinical, endoscopic, and histopathological characteristics of the enrolled cohort. FIT-positive individuals referred for screening colonoscopy at participating Italian centers were screened for eligibility, underwent colonoscopy, and were invited to provide a stool sample for microbiome analysis. A total of 315 individuals were screened, of whom 212 (67%) were enrolled. Among eligible patients, 90% agreed to enroll after receiving study information. Overall, 200 (94%) of enrolled individuals completed the required study activities, including stool sample collection and colonoscopy, indicating high compliance with study procedures. Colonoscopy was performed in 209 patients (99% of enrolled patients). CRC was detected in 7 patients (3%), and advanced colorectal adenomas in 39 (18%), while 86 (41%) colonoscopies were negative. The positive predictive value of FIT was 3.35% for CRC and 18.66% for advanced adenomas. In our preliminary analysis, patient acceptance and compliance with microbiome-based sampling were high among FIT-positive individuals undergoing CRC screening. These findings support the feasibility of conducting microbiome-based studies within organized screening programs. Analyses aimed at developing and validating the microbiome-based diagnostic tool are currently ongoing and are beyond the scope of the present report.

## 1. Introduction

Colorectal cancer (CRC) represents one of the most common malignancies globally: it occupies the third position in global cancer incidence and is the second leading cause of cancer-related death [1]. In addition to its clinical implications, CRC exerts a considerable economic impact on healthcare systems, owing to the cumulative costs of hospitalization, therapeutic interventions, surgical procedures, and palliative management [2].

The etiologic landscape of CRC encompasses both immutable and potentially modifiable determinants [3]. Non-modifiable contributors include male sex, older age, genetic susceptibility and certain pre-existing medical conditions. In contrast, lifestyle behaviors and dietary habits constitute major modifiable contributors and play a key role in colorectal carcinogenesis [4].

Extensive research over the past dees has established a key role for the gut microbiome in CRC development and progression [4,5]. CRC patients consistently show dysbiosis, marked by the overgrowth of pro-tumorigenic taxa such as *Fusobacterium nucleatum* and *Parvimonas micra*, which promote inflammatory or genotoxic environments, alongside a reduction in protective, short chain fatty acid producing bacteria [6]. More recently, increasing evidence has suggested that not all *Fusobacterium nucleatum* strains exhibit the same biological behavior: distinct subspecies and phylogenetic lineages appear to differ in their capacity to colonize colorectal tumors, promote carcinogenesis, and influence disease progression [7].

Population-based screening for average-risk individuals effectively decreases CRC incidence and mortality by enabling early detection [8]. The primary objective is the identification and removal of colorectal adenomas and sessile serrated lesions, thereby preventing malignant progression. An optimal screening test should achieve high diagnostic accuracy while remaining non-invasive, safe, feasible, and affordable [8].

CRC screening options include invasive and non-invasive methods, each with distinct advantages and limitations. While colonoscopy and flexible sigmoidoscopy allow direct lesion removal and histological assessment, their cost, invasiveness, and resource demands limit widespread use [9]. Non-invasive tests such as FIT are more accessible but have reduced sensitivity for advanced neoplasia and still require confirmatory colonoscopy after a positive result [8,9].

In Italy, organized CRC screening programs are based on a biennial FIT offered to average-risk individuals aged 50–69 years, with several regions extending the target age up to 74 years. Individuals with a positive FIT result are referred for diagnostic colonoscopy, which remains the reference standard for the detection of CRC and advanced colorectal adenomas. This two-step approach aims to balance screening effectiveness, patient acceptability, and healthcare resource utilization [10,11].

Despite its established role, FIT is intrinsically based on the detection of occult bleeding, a phenomenon that generally occurs relatively late during colorectal carcinogenesis. Consequently, lesions that bleed intermittently or minimally, particularly advanced adenomas, may remain undetected. This limitation has stimulated extensive research into complementary biomarkers capable of identifying biological alterations associated with neoplastic transformation before the onset of clinically detectable bleeding [12,13].

Among non-invasive screening approaches, microbiome-based diagnostic tools have gained increasing prominence in recent years. Advances in sequencing technologies have identified microbial signatures that distinguish CRC from healthy individuals [4], as well as the development of machine-learning models integrating fecal microbiome profiles to improve diagnostic accuracy across different populations [14,15]. Recent studies have demonstrated that these approaches can achieve promising diagnostic performance while maintaining good discrimination in independent validation cohorts, supporting their potential as complementary tools to current FIT-based screening strategies. These advances have also led to the development of targeted microbial assays, such as the qPCR-based 4Bac panel for detecting CRC and advanced colorectal adenomas [16].

Although international guidelines acknowledge the potential of microbiome-derived biomarkers for CRC detection, they also emphasize that current evidence remains insufficient to support their routine implementation in screening programs because of limited methodological standardization, restricted assay availability, substantial inter-individual variability, and the need for multicenter external validation and demonstration of clinical utility [17]. Harmonization of sample collection procedures, sequencing methodologies, and bioinformatic pipelines has therefore been identified as a key prerequisite for ensuring reproducibility and facilitating the future clinical implementation of microbiome-based diagnostic strategies [17].

A pivotal determinant of successful CRC screening is represented by patient’s autonomy of choice [18,19,20]. Contemporary guidelines explicitly endorse offering a spectrum of screening modalities, underscoring that the decision should be shaped by patient preferences rather than clinician-driven selection [18,19,20]. International recommendations urge healthcare providers to actively support individualized choice when discussing screening options [21]. A substantial body of evidence shows that empowering individuals to choose, or at least to receive a sequential offer of different tests, significantly enhances participation: these findings highlight that respecting patient choice is not merely an ethical imperative but a practical strategy to improve screening uptake [22].

Within this framework, a central challenge in implementing microbiome-based diagnostic and therapeutic tools concerns how both clinicians and patients perceive and engage with these emerging approaches. Current evidence shows that, although clinicians express strong interest in microbiome applications, their expertise and confidence in interpreting microbiome-derived data remain limited [23,24]. On the other hand, patients tend to be highly receptive to microbiome-based interventions, yet both groups require structured education and clear, evidence-based frameworks to ensure safe and appropriate clinical adoption [23,24]. Studies assessing attitudes toward microbiome-based tools highlight this dual dynamic. Surveys show that patients generally view microbiome research positively and are willing to provide stool samples—often for altruistic reasons—though practical issues such as home sample storage may hinder participation. Clear explanations of procedures have been shown to markedly improve acceptance and compliance [25]. Clinicians, by contrast, often lack adequate training to interpret microbiome data, and expert consensus repeatedly emphasizes the need for targeted educational initiatives to enable appropriate clinical use [23].

Building on this foundation, we designed a multicenter, prospective observational study involving patients in the national CRC screening program, aimed at developing a non-invasive, straightforward, interpretable gut microbiome-based diagnostic tool for identifying CRC and advanced colorectal adenomas. The parent study seeks to improve risk stratification among FIT-positive individuals and ultimately enhance the performance of currently available non-invasive CRC screening strategies, optimizing colonoscopy referral pathways. The microbiome analyses required for the development and validation of the diagnostic model are still ongoing and are not the subject of the present report. At the same time, despite the growing interest in microbiome-based approaches for CRC detection, limited evidence is available regarding patient willingness to participate in microbiome-based studies and to comply with stool collection procedures required for microbiome analyses. Therefore, this preliminary analysis was specifically designed to investigate patient acceptance and compliance with participation in a microbiome-based study, as well as the broader willingness of individuals undergoing CRC screening to engage with diagnostic approaches based on gut microbiota profiling. Understanding these aspects is essential for the future implementation of microbiome-based screening strategies, regardless of their diagnostic performance.

## 2. Materials and Methods

### 2.1. Study Design and Population

The present study describes the preliminary data from an observational prospective multicenter study aimed at developing a gut microbiome-based diagnostic tool to improve the detection of CRC and advanced colorectal adenomas (PNRR-POC-2023-12377319). The planned study duration is 24 months, and the present manuscript reports a preliminary analysis of data collected during the ongoing study. Microbiome sequencing analyses, biomarker identification, and the development and validation of the diagnostic model will be performed after completion of patient recruitment and are beyond the scope of the present report.

The primary objectives of this preliminary analysis were to evaluate patient acceptance of participation in the study and compliance with the required study procedures, thereby assessing the feasibility of conducting microbiome-based research within an organized CRC screening program. Secondary objectives included describing the reasons for non-enrollment together with the demographic, clinical, endoscopic, and histopathological characteristics of the enrolled cohort. These data were collected to provide a comprehensive characterization of the study population and to establish the clinical framework for the subsequent development and validation of microbiome-based diagnostic models planned in the parent study.

Participants were selected among FIT-positive individuals participating in the Italian national colorectal cancer screening program and referred for screening colonoscopy at the participating study centers: Fondazione Policlinico Universitario Agostino Gemelli IRCCS (Rome), Fondazione Poliambulanza Istituto Ospedaliero (Brescia), and Fondazione IRCCS Casa Sollievo della Sofferenza (San Giovanni Rotondo). To be eligible for enrollment, patients were required to meet the following criteria: age 50–74 years, a positive FIT result and the ability to provide written informed consent. Exclusion criteria included inability to undergo colonoscopy, a current diagnosis of another malignancy, the presence of severe comorbidities or organic gastrointestinal diseases (e.g., diverticular disease, inflammatory bowel disease), and the use of antibiotics, probiotics, or proton pump inhibitors within 4 weeks before enrollment.

This study was reported in accordance with the Strengthening the Reporting of Observational Studies in Epidemiology (STROBE) statement, and a completed STROBE checklist is provided as Supplementary Materials. Informed consent was obtained from all subjects involved in the study.

### 2.2. Study Procedures

The screening visit was conducted to assess eligibility criteria (inclusion and exclusion) in FIT-positive individuals participating in the regional colorectal cancer screening program, and to record the reasons for study exclusion. During the same visit, eligible participants received detailed information regarding the study objectives, stool sample collection procedures, and the subsequent study workflow before providing written informed consent.

At baseline, we collected the following demographic and clinical data in all enrolled patients: personal and family history of CRC, comorbidities, current medications, and the presence of gastrointestinal or alarm symptoms such as iron-deficiency anemia, hematochezia, unexplained weight loss, abdominal pain, or recent changes in bowel habits. Data on dietary patterns, smoking status, alcohol consumption, body mass index (BMI), and any previous FIT or colonoscopy reports (including virtual colonoscopy) were also collected. During the same visit, all enrolled participants received a standardized stool collection kit containing a nucleic acid preservative (Prebiomics srl, Trento, Italy), together with instructions describing the collection procedure.

Stool samples were collected before bowel preparation for colonoscopy without any study-mandated dietary modifications or additional interventions. Samples were stored at −80 °C at each participating center immediately after delivery and were subsequently shipped on dry ice to the University of Trento, where DNA extraction and shotgun metagenomic sequencing will be performed. Sequencing quality control, bioinformatic analyses, biomarker development, and validation procedures will be described in detail in future reports focused specifically on microbiome-derived outcomes.

All enrolled participants subsequently underwent colonoscopy within four weeks from enrollment according to routine clinical practice within the Italian organized CRC screening program. Colonoscopies were performed with high-definition endoscopes by expert endoscopists accredited for participation in the national screening program, with >5000 total colonoscopies and at least 300 in the last year, in accordance with the accreditation requirements of the national screening program. Histology of resected lesions was assessed by experienced pathologists according to the WHO classification and the Vienna criteria [26,27]. Advanced colorectal adenomas have been defined as adenomas larger than or equal to 10 mm, and/or with villous components higher than or equal to 25%, and/or high-grade dysplasia.

All demographic, clinical, endoscopic, histopathological, and study-related information was prospectively entered into a dedicated electronic Case Report Form (eCRF) using the REDCap platform (v14.0.10). The use of a shared electronic case report form and standardized operating procedures across participating centers was intended to minimize inter-center variability in patient assessment, biological sample collection, and clinical data recording.

### 2.3. Statistical Analysis

Acceptance was defined as the willingness of eligible individuals to participate in the study and was assessed by recording the proportion of eligible subjects who agreed to enroll after receiving study information. Compliance was defined as adherence to the study procedures and was assessed by recording completion of the required study activities within enrolled patients, including stool sample collection and colonoscopy.

Continuous variables were summarized as mean values, with corresponding measures of dispersion reported where available, whereas categorical variables were summarized as absolute frequencies and percentages. Reasons for non-enrollment, demographic characteristics, clinical variables, endoscopic findings, and histopathological outcomes were analyzed descriptively.

As an exploratory analysis, the positive predictive value (PPV) of FIT for CRC and advanced colorectal adenomas was calculated using the total number of FIT-positive individuals who underwent colonoscopy within the study cohort as the denominator and the number of subjects with the target lesion confirmed by colonoscopy and histopathology as the numerator. Proportions were reported together with their corresponding 95% confidence intervals (95% CIs), calculated using the exact binomial (Clopper–Pearson) method.

Given the preliminary nature of the present analysis and the absence of microbiome-derived diagnostic results, no inferential statistical analyses or multivariable models were performed.

## 3. Results

### 3.1. Study Population

**Figure 1:**
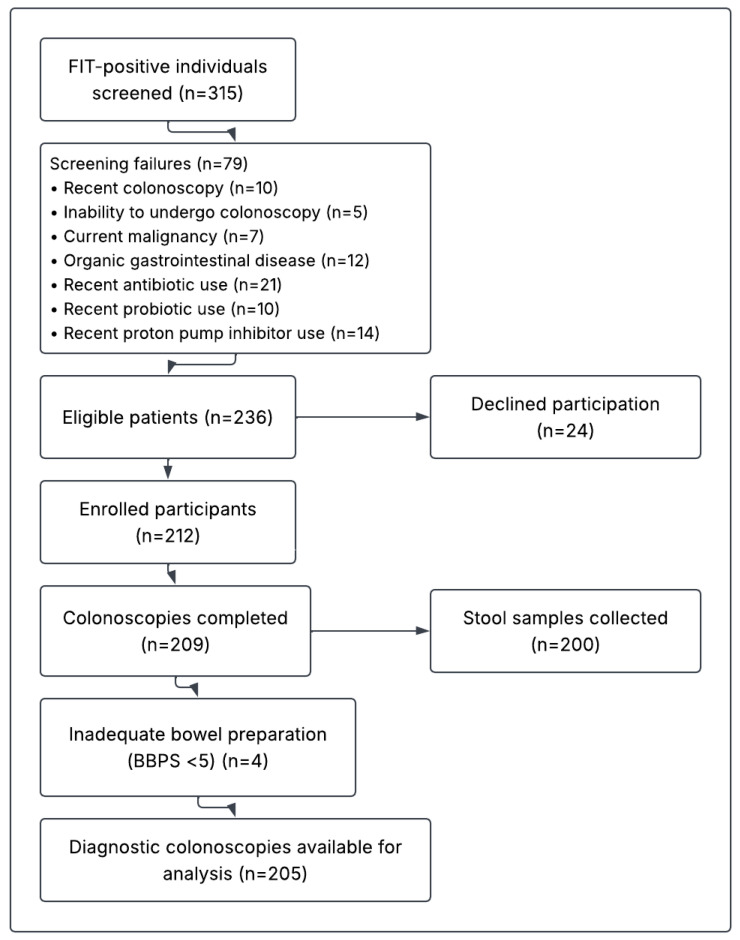
STROBE flow diagram of participant selection and study procedures.

Between 29 November 2024 and 1 December 2025, a total of 315 patients attended the screening visit at the Digestive Endoscopy Unit of the Gemelli University Hospital (Rome), Fondazione Poliambulanza Hospital (Brescia) and Casa Sollievo della Sofferenza Hospital (San Giovanni Rotondo). Of them, 212 (67%) were ultimately enrolled, while 103 (33%) were not enrolled after the screening visit. Notably, 236 patients (75%) met all inclusion criteria and did not present any exclusion criteria. Among this eligible subgroup, 24 (10%) declined participation after receiving study information, resulting in an overall acceptance rate of 89.8% (95% CI 85.2–93.4%). Among enrolled patients, 200 (94%) successfully completed stool sample collection for metagenomic analysis according to the study protocol. A total of 12 protocol deviations (drop-out) were recorded during follow-up, including 3 participants who did not undergo the scheduled colonoscopy and 9 who underwent colonoscopy but did not provide the stool sample required by the study protocol. Consequently, the vast majority of enrolled participants successfully completed all study procedures according to protocol, resulting in an overall compliance rate of 94.3% (200/212; 95% CI 90.3–97.0%). The overall process of participant selection, enrollment, and completion of the study procedures is summarized in the flow diagram shown in Figure 1. An overview of the reasons for exclusion of screened patients is reported in Table 1.

### 3.2. Clinical Characteristics of the Study Population

Among enrolled patients, 97 (46%) were female, with a mean age of 59.3 ± 5.9 years and a mean Charlson Comorbidity Index (CCI) of 1.75 ± 0.82. No enrolled patients had a personal history of CRC, whereas 37 patients (18%) reported a family history of the disease. Gastrointestinal or alarm symptoms were present in 69 individuals (32%), including hematochezia in 26 patients (12%), abdominal pain in 23 (11%), iron-deficiency anemia in 9 (4%), recent changes in bowel habits in 7 (3%), and unexplained weight loss in 2 (1%). Lifestyle-related characteristics, including smoking habits, alcohol consumption, dietary patterns, and body mass index, were also systematically recorded. Most participants reported consuming fruit and vegetables at least once daily, whereas overweight or obesity (BMI ≥ 25 kg/m^2^) was observed in 129 participants (61%). Smoking habits and alcohol consumption showed a heterogeneous distribution across the study population.

A detailed overview of the demographic, clinical, and lifestyle characteristics of the study population is provided in Table 2.

### 3.3. Endoscopic Features of the Study Population

Among enrolled participants, 209 (99%) underwent colonoscopy within four weeks of receiving a positive FIT result. A total of 4 (2%) procedures were deemed non-diagnostic due to inadequate bowel preparation (Boston Bowel Preparation Scale, BBPS < 5), whereas adequate bowel cleansing (BBPS 6–9) was achieved in 205 patients (98%).

At the patient level, 86 (41%) colonoscopies were negative, with no significant lesions identified, while at least one colorectal lesion was detected in 123 participants (59%). Seven (3.35%; 95% CI 1.4–6.8%) patients were diagnosed with CRC and 39 (18.66%; 95% CI 13.6–24.6%) with advanced colorectal adenoma. The remaining 77 (36.8%) participants presented other lesions, including non-advanced adenomas, hyperplastic polyps, serrated lesions, or other benign findings not fulfilling the criteria for CRC or advanced colorectal adenoma.

At the lesion level, a total of 264 lesions were identified and histopathologically characterized. Most lesions measured less than 10 mm (214 lesions, 81%), whereas 33 (13%), 12 (5%), and 5 (2%) measured >10 mm, >20 mm, and >30 mm, respectively. Lesions were distributed throughout the colon, involving the right colon (92 lesions, 35%), transverse colon (55 lesions, 21%), left colon (77 lesions, 29%), and rectum (40 lesions, 15%). According to the WHO classification, the majority of lesions were adenomas (201/264, 76%), while 39 (15%) were hyperplastic lesions and 24 (9%) were serrated lesions. According to the Vienna classification, most lesions showed low-grade dysplasia (196/264, 74%), whereas 10 (4%) showed high-grade dysplasia and 7 (3%) were invasive carcinomas.

Detailed endoscopic and histopathological findings, including lesion size, location, morphology, and corresponding tissue characteristics, are summarized in Table 3.

### 3.4. Association Between Colonoscopy Outcomes and FIT Results

In the study population, which comprised solely FIT-positive patients, the test showed a positive predictive value of 3.35% (95% CI 1.4–6.8%) for CRC detection. Regarding advanced colorectal adenomas, the positive predictive value was 18.66% (95% CI 13.6–24.6%).

## 4. Discussion

Beyond diagnostic accuracy, the implementation of any novel screening strategy depends on its acceptability by the target population and on the practical feasibility of integrating it into existing healthcare pathways.

In the case of microbiome-based approaches, additional logistical steps—including stool collection outside the hospital setting, sample preservation, transportation, and processing—may represent potential barriers to patient participation. Concerns regarding stool handling, storage, or the perceived complexity of sample collection could reduce adherence if not adequately addressed. Therefore, demonstrating that these procedures are well accepted by participants represents an essential prerequisite for the future clinical evaluation of microbiome-derived biomarkers.

In this preliminary multicenter analysis of a prospective study conducted in a FIT-positive population, we observed high levels of patient acceptance and compliance with microbiome-based sampling, alongside endoscopic findings consistent with real-world CRC screening data.

The proportion of patients declining participation was low (10%), further supporting the acceptability of this approach in a real-world setting. These findings indicate that microbiome-based stool sampling is well accepted among FIT-positive individuals participating in an organized CRC screening program.

Compliance with study procedures was high, with 94% of enrolled participants successfully providing a sample. This observation is consistent with prior evidence indicating that patients are generally receptive to microbiome-based strategies, particularly when non-invasive and supported by clear instructions. Overall, our findings suggest that the potential logistical challenges associated with stool collection for microbiome analyses can be effectively managed within an organized screening program, supporting the practical feasibility of incorporating stool sample collection into microbiome-based research protocols conducted in real-world clinical settings.

From a diagnostic standpoint, the positive predictive values observed for FIT (3.35% for CRC and 18.66% for advanced colorectal adenomas) are in line with those reported in population-based screening programs, confirming the representativeness and external validity of our cohort. This observation supports the appropriateness of the study population for the parent project, whose objective is the development and validation of microbiome-derived biomarkers in a real-world FIT-positive screening setting.

This study has several strengths, including its multicenter design and its implementation within an organized population-based CRC screening program involving multiple referral centers. This real-world setting allowed the evaluation of patient acceptance and compliance under routine clinical conditions rather than in a highly selected research environment. Furthermore, the prospective collection of comprehensive clinical, endoscopic, histopathological, and biological data provides a robust foundation for the subsequent development, training, and validation of microbiome-based diagnostic models within the parent study.

However, some limitations should be acknowledged. First, this is a preliminary analysis, and the sample size may limit more detailed subgroup evaluations. In addition, the study population consisted exclusively of FIT-positive individuals who had already agreed to participate in an organized CRC screening program and were referred for colonoscopy. Consequently, the observed acceptance and compliance rates may not be generalizable to the broader screening population or to individuals offered microbiome-based testing as a first-line screening strategy: participants enrolled in organized screening programs may be inherently more motivated and health-conscious than the general population, potentially leading to an overestimation of acceptance and adherence to study procedures. Third, although high rates of stool sample collection support the feasibility of conducting microbiome-based studies within organized screening programs, these findings alone do not demonstrate the feasibility of integrating microbiome-based approaches into routine clinical practice. Future implementation will also depend on diagnostic performance, reproducibility, cost-effectiveness, standardization, and clinical utility. Finally, microbiome sequencing analyses, biomarker identification, and diagnostic model development are still ongoing. Therefore, no conclusions can currently be drawn regarding the diagnostic accuracy or clinical performance of the proposed microbiome-based tool, which will be evaluated and reported separately in the parent study.

## 5. Conclusions

In conclusion, microbiome-based sampling showed high acceptance and compliance among FIT-positive individuals participating in an organized CRC screening program. These findings indicate that the collection of stool samples for microbiome analyses can be successfully integrated into the routine workflow of population-based screening programs, representing an important prerequisite for the future clinical evaluation of microbiome-derived biomarkers.

Although the diagnostic performance of the proposed microbiome-based tool remains to be established after completion of the parent study, our findings suggest that patient participation and adherence are unlikely to represent major barriers to its future implementation. Further analyses integrating microbiome sequencing data with the comprehensive clinical, endoscopic, and histopathological information collected in this cohort will determine whether microbiome-based biomarkers can meaningfully improve current CRC screening strategies and optimize risk stratification among FIT-positive individuals.

## Figures and Tables

**Table 1:** Reasons for non-enrollment. Percentages were calculated relative to the total number of individuals who were not enrolled in the study (*n* = 103).

Reason for Exclusion	Number of Patients (%)
Patient refusal	24 (23%)
Recent colonoscopy	10 (10%)
Inability to undergo colonoscopy	5 (5%)
Current diagnosis of another malignancy	7 (7%)
Presence of organic gastrointestinal diseases	12 (12%)
Use of antibiotics within the 4 weeks preceding enrollment.	21 (20%)
Use of probiotics within the 4 weeks preceding enrollment.	10 (10%)
Use of proton pump inhibitors within the 4 weeks preceding enrollment.	14 (13%)

**Table 2:** Enrolled population characteristics (CCI = Charlson comorbidity index; BMI = Body Mass Index; CRC = colorectal cancer; SD standard deviation).

Demographic Characteristics		
Female	97 (46%)	
Age (mean ± SD)	59.3 ± 5.9 years	
Personal history of CRC	0 (0%)	
Family history of CRC	37 (18%)	
CCI (mean ± SD)	1.75 ± 0.82	
**Alarm symptoms**		
Iron-deficiency anemia	9 (4%)	
Hematochezia	26 (12%)	
Unexplained weight loss	2 (1%)	
Abdominal pain	23 (11%)	
Recent changes in bowel habits	7 (3%)	
**Risk factors**		
Consumption of red meat	<1/week	18 (9%)
	1/week	129 (61%)
	2/week	35 (16%)
	>2/week	30 (14%)
Consumption of fruit and vegetables	Less than once/day	1 (1%)
	1/day	85 (40%)
	2/day	78 (37%)
	>2/day	47 (22%)
Smoking habit	Active	48 (23%)
	Former	15 (7%)
Alcohol consumption	None	116 (55%)
	Occasional	69 (32%)
	Daily	27 (13%)
BMI	<18	1 (1%)
	18–25	82 (39%)
	25–30	85 (40%)
	>30	44 (21%)

**Table 3:** Endoscopic and histopathological findings (BBPS = Boston Bowel Preparation Scale; WHO = World Health Organization; LST-G = Laterally Spreading Tumor—Granular type; LST-NG = Laterally Spreading Tumor—Non-Granular type; CRC = colorectal cancer; CAD = advanced colorectal adenoma). Other lesions include non-advanced adenomas, hyperplastic polyps, serrated lesions, and other non-malignant findings not meeting the criteria for advanced colorectal adenomas or CRC.

Endoscopic Outcomes 209 Patients/Colonoscopies		
BBPS	<3	2 (1%)
	3–5	2 (1%)
	6–9	205 (98%)
Number of lesions (single colonoscopy)	0	86 (41.2%)
	1–4	114 (54.5%)
	>4	9 (4.3%)
Outcome	Negative	86 (41.1%)
	CAD	39 (18.7%)
	CRC	7 (3.3%)
	Other lesions	77 (36.8%)
**Histopathological findings** ***n* = 264 lesions**		
Lesion size	<10 mm	214 (81.1%)
	>10 mm	33 (12.5%)
	>20 mm	12 (4.5%)
	>30 mm	5 (1.9%)
Lesion location	Right	92 (34.8%)
	Transverse	55 (20.8%)
	Left	77 (29.2%)
	Rectum	40 (15.2%)
Lesion morphology (Paris classification)	0-Is	174 (65.9%)
	0-Ip	48 (18.2%)
	0-Isp	0 (0%)
	0-IIa	25 (9.5%)
	0-IIb	11 (4.2%)
	0-IIc	0 (0%)
	0-III	3 (1.1%)
	LST-G	3 (1.1%)
	LST-NG	0 (0%)
**Histopathological findings**		
Tissue characteristics (WHO classification)	Hyperplastic	39 (14.8%)
	Adenoma	201 (76.1%)
	Serrated	24 (9.1%)
Dysplasia (Vienna classification)	No dysplasia	51 (19.3%)
	Undefined	0 (0%)
	Low grade	196 (74.2%)
	High grade	10 (3.8%)
	Invasive	7 (2.7%)
	carcinoma	

## Data Availability

The data presented in this study are available on request from the corresponding author due to privacy and ethical restrictions.

## Supplementary Materials

The following supporting information can be downloaded at: https://www.mdpi.com/article/10.3390/microorganisms14071564/s1, File S1: strobe checklist + consent.
