# Supplementary material for: Feasibility and Compliance of Stool Collection for Future Microbiome-Based Colorectal Cancer Screening: Preliminary Findings from a Prospective Multicenter FIT-Positive Cohort
Source: Microorganisms. 2026 Jul 17;14(7):1564. doi: 10.3390/microorganisms14071564 (PMC13413819; doi:10.3390/microorganisms14071564)
Supplement: Supplementary file 1 [file microorganisms-14-01564-s001.zip › microorganisms-4395538-supplementary.pdf]

**FOGLIO INFORMATIVO PER LA PARTECIPAZIONE AD UNO STUDIO OSSERVAZIONALE E DICHIARAZIONE DI CONSENSO PER SOGGETTI CAPACI**

11.11.2025 versione 2.0

**SCHEDA INFORMATIVA**

Gentile Signora/e,

Presso la struttura Fondazione Policlinico Universitario Agostino Gemelli IRCCS) è in programma una ricerca medico-scientifica dal titolo ‘’**Sviluppo di uno strumento diagnostico non invasivo basato sul microbiota intestinale per lo screening del cancro del colon-retto (Studio NI-GUILTI)**’’.

Questa ricerca è a carattere nazionale–multicentrica.

Per svolgere tale ricerca abbiamo bisogno della collaborazione e disponibilità di persone che, come Lei, soddisfano i requisiti scientifici idonei alla valutazione che verrà eseguita. Comunque, prima che Lei prenda la decisione di accettare o rifiutare di partecipare, La preghiamo di leggere con attenzione, questo documento prendendo tutto il tempo necessario e di chiederci chiarimenti qualora non comprendesse o avesse bisogno di ulteriori precisazioni. Inoltre, qualora lo desiderasse, prima di decidere, può chiedere un parere ai Suoi familiari o ad un Suo medico di fiducia.

**CHE COSA SI PROPONE LO STUDIO**

Numerose evidenze dimostrano che le alterazioni del microbioma intestinale sono coinvolte nella patogenesi del tumore del colon-retto (CRC). Il microbiota intestinale è l'insieme dei microrganismi presenti nell'intestino mentre per microbioma s'intende il patrimonio genetico del microbiota, cioè a tutto il DNA e RNA dei microrganismi. Inoltre, studi recenti hanno individuato signature microbiche in grado di discriminare tra pazienti con CRC e controlli sani. Sulla base di queste evidenze, le linee guida internazionali hanno recentemente sostenuto l'utilizzo di biomarcatori basati sul microbioma per lo screening del CRC, ma tali studi non sono ad oggi ancora disponibili. Studiando il microbioma intestinale in un'ampia coorte di pazienti FIT (test immunochimico fecale)-positivi sottoposti a colonscopia di screening, miriamo a sviluppare e brevettare uno strumento diagnostico basato sul microbioma intestinale con lo scopo di migliorare la diagnosi di CRC e degli adenomi colorettali avanzati. In particolare, la partecipazione allo studio è riservata a pazienti che soddisfano i criteri di inclusione previsti, ovvero: adesione al programma nazionale di screening del cancro del colon-retto (età compresa tra 50 e 74 anni), esito positivo del test FIT e capacità di fornire il consenso informato scritto e di rispettare le procedure dello studio.

Lo studio ha come obiettivo generale quello di identificare specifici biomarcatori microbici associati al tumore del colon-retto (CRC) e sviluppare un nuovo strumento diagnostico, basato sul microbioma intestinale e sulle sue caratteristiche funzionali, in grado di fornire una diagnosi precoce del CRC.

In particolare, con la ricerca che qui presentiamo, si intendono ottenere dati relativi alla caratterizzazione del microbiota intestinale di tutti i pazienti arruolati al fine di identificare profili microbici associati a una diagnosi precoce del tumore del colon-retto.

Obiettivi:

1. Reclutare una coorte di pazienti FIT positivi, raccogliere dati clinici e campioni di feci ed eseguire la colonscopia nei soggetti arruolati.
2. Caratterizzare in modo completo il microbioma intestinale dei soggetti arruolati.

3. Valutare la performance del FIT nel predire la presenza di adenomi avanzati o CRC.
4. Identificare specifici biomarcatori microbici associati al CRC.

Sviluppare un nuovo strumento diagnostico, basato sul microbioma e sulle sue caratteristiche funzionali, in grado di fornire una diagnosi precoce del CRC.

**COSA COMPORTA LA SUA PARTECIPAZIONE ALLO STUDIO**

Se accetta di partecipare a questo studio Lei sarà sottoposto/a ad una prima visita per verificare che le sue condizioni soddisfino i criteri richiesti dallo studio.

Lo studio prevede:

- Raccolta dei dati clinici e dei campioni fecali. I dati clinici saranno raccolti durante la prima valutazione clinica di arruolamento. Successivamente, le chiederemo di fornirci un campione fecale in un apposito kit che le consegneremo entro 2 settimane dall'arruolamento; il campione sarà congelato e conservato in Fondazione Policlinico Universitario Agostino Gemelli IRCCS fino al momento dell'invio all'Università di Trento dove verrà eseguita l'analisi del microbioma;
- I dati raccolti durante lo studio saranno registrati in un modulo elettronico sulla piattaforma REDCap, un sistema informatico sicuro che permette di conservare e proteggere le informazioni dei partecipanti.
- Verrà eseguita una colonscopia entro 4 settimane dalla positività del FIT come da pratica clinica presso la Fondazione Policlinico Universitario Agostino Gemelli IRCCS. I pazienti FIT positivi saranno sottoposti a colonscopia come da pratica clinica anche se non accettano di partecipare al presente studio;

- Entro 2 settimane dalla colonscopia verranno raccolti i dati endoscopici e istopatologici come da pratica clinica;
- Al termine della fase di arruolamento, effettueremo l'analisi del microbioma e svilupperemo uno strumento diagnostico basato sul microbioma intestinale per l'identificazione di CRC e adenomi colorettali avanzati. Tale analisi verrà eseguita presso il Laboratorio di metagenomica computazionale di Trento.

Lo studio durerà 24 mesi e parteciperanno a questa ricerca 1006 pazienti di cui circa 400 verranno presso la Fondazione Policlinico Universitario Agostino Gemelli IRCCS oltre che negli altri centri coinvolti nello studio.

**QUALI SONO I RISCHI DERIVANTI DALLA PARTECIPAZIONE ALLO STUDIO**

La partecipazione allo studio non prevede l'esecuzione di indagini o trattamenti diversi rispetto a quelli previsti nella normale pratica clinica e pertanto nello studio non ci saranno rischi aggiuntivi rispetto alla pratica clinica.

**QUALI SONO I BENEFICI CHE POTRÀ RICEVERE PARTECIPANDO ALLO STUDIO**

Dalla partecipazione a questo studio non sono prevedibili benefici diretti per Lei, ma la sua partecipazione ci consentirà di acquisire informazioni aggiuntive circa la patologia da cui Lei è affetto/a.

**COSA SUCCEDE SE DECIDE DI NON PARTECIPARE ALLO STUDIO**

Lei è libero/a di non partecipare allo studio. In questo caso riceverà, comunque, tutte le terapie standard previste per la Sua patologia, senza alcuna penalizzazione, ed i medici continueranno a seguirla comunque con la dovuta attenzione assistenziale.

**INTERRUZIONE DELLO STUDIO**

La Sua adesione a questo programma di ricerca è completamente volontaria e Lei si potrà ritirare dallo studio in qualsiasi momento dandone comunicazione allo Sperimentatore. In questo caso i dati

raccolti fino al momento del ritiro saranno considerati nei risultati in forma aggregata ed anonima per l'analisi finale.

**INFORMAZIONI CIRCA I RISULTATI DELLO STUDIO**

Se Lei lo richiederà, alla fine dello studio potranno esserLe comunicati i risultati dello studio in generale ed in particolare quelli che La riguardano.

**ULTERIORI INFORMAZIONI**

Per ulteriori informazioni e comunicazioni durante lo studio sarà a disposizione il seguente personale: Dr. Gianluca Ianiro - gianluca.ianiro@unicatt.it tel. 063015 9539

*Il protocollo dello studio che Le è stato proposto è stato esaminato ed approvato dal Comitato Etico Territoriale (CET) Lazio Area 3. Il CET ha tra le altre cose verificato la conformità dello studio alle Norme di Buona Pratica Clinica ed ai principi etici espressi nelle Dichiarazione di Helsinki e che la sicurezza, i diritti e il vostro benessere siano stati protetti.*

*Qualora ritenesse opportuno segnalare eventi o fatti relativi allo studio cui ha aderito a soggetti non direttamente coinvolti nello studio stesso potrà fare riferimento al CET che ha approvato lo studio (INDICARE).*

## DICHIARAZIONE DI CONSENSO

(*questa dichiarazione deve essere firmata e datata personalmente dal paziente e dal medico che ha condotto la discussione relativa al consenso informato*)

**DICHIARO**

◯ di aver ricevuto dal Dottor ______________________________________________ esaurienti spiegazioni in merito alla richiesta di partecipazione alla ricerca in oggetto, secondo quanto riportato nella sezione informativa della quale mi è stata data prima d'ora una copia, facente parte di questo consenso, della quale mi è stata consegnata una copia in data _____________

◯ che mi sono stati chiaramente spiegati ed ho compreso la natura, le finalità, le procedure, i benefici attesi, i rischi e gli inconvenienti possibili;

◯ di aver avuto l'opportunità di porre qualsivoglia domanda allo sperimentatore dello studio e di aver avuto risposte soddisfacenti;

◯ di aver avuto il tempo sufficiente per riflettere sulle informazioni ricevute;

◯ di avere avuto il tempo sufficiente per discuterne con terzi;

◯ di essere consapevole che la ricerca potrà essere interrotta in ogni momento;

◯ di essere stato informato che i risultati dello studio saranno resi noti alla comunità scientifica, tutelando la mia identità secondo la normativa vigente sulla privacy;

◯ di essere consapevole che qualsiasi scelta espressa in questo modulo di consenso potrà essere revocata in qualsiasi momento e senza alcuna giustificazione;

◯ di aver ricevuto una copia del presente modulo di consenso.

__________________ __________________________________________________

Data Firma del paziente

Data Firma del medico che ha informato il paziente

(*Se il paziente non è in grado di leggere o di firmare, un* <u>*testimone indipendente*</u> *dallo sperimentatore e dallo sponsor deve essere presente durante l'intera discussione relativa al consenso informato. Il testimone deve firmare e datare personalmente la dichiarazione di consenso informato dopo che il modulo stesso e qualsiasi altra informazione scritta siano stati letti e spiegati al soggetto e questi abbia espresso il consenso verbale alla partecipazione allo studio*).

In questo caso:

Io sottoscritto ………………………………. testimonio che il dottor …………………………………………………………………ha esaurientemente spiegato al Sig. ……………………………………………………………….
le caratteristiche dello studio in oggetto, secondo quanto riportato nella scheda informativa qui allegata, e che lo stesso, avendo avuto la possibilità di fare tutte le domande che ha ritenuto necessarie, ha accettato liberamente di aderire allo studio.

Data…………… Firma del testimone indipendente …………………………………….

Data…………… Firma del medico che ha dato le informazioni al paziente ………………………………

Gemelli

Fondazione Policlinico Universitario Agostino Gemelli IRCCS
Università Cattolica del Sacro Cuore

# InformativaeConsensoperilTrattamentodeiDatiPersonaliperFinalitàdiRicercaScientifica perAdulti
(Art.13e14delGDPREU2016/679)

| | |
|---|---|
| **Promotore:** | **Fondazione Policlinico Universitario Agostino Gemelli IRCCS Largo Francesco Vito, n. 1 – 00168 – Roma** (nel seguito **"Fondazione")** |
| **Nome dello Studio:** | **''Sviluppo di uno strumento diagnostico non invasivo basato sul microbiota intestinale per lo screening del cancro del colon-retto'' - PNRR-POC-2023-12377319 (Studio NI-GUILTI)** |
| **Sperimentatore principale del Promotore** | Dr. Gianluca Ianiro UOC Gastroenterologia |

Versione 1.0 data 26/06/2024

La Fondazione, in qualità di Titolare autonomo del Trattamento:

- in accordo alle responsabilità previste dalle norme di Buona Pratica Clinica (D.L. 211/2003);
- in ottemperanza alle disposizioni:
  - ✓ del Regolamento UE 2016/679 del Parlamento e del Consiglio Europeo relativo alla protezione delle persone fisiche con riguardo al trattamento dei dati personali, nonché alla libera circolazione di tali dati (di seguito GDPR EU 2016/679);
  - ✓ del D.Lgs. 30 giugno 2003, n. 196 così come integrato con le modifiche introdotte dal D.Lgs. 10 agosto 2018, n.101;
  - ✓ del Provvedimento dell'Autorità Garante recante le prescrizioni relative al trattamento di categorie particolari di dati, ai sensi dell'art. 21 comma 1 del D.Lgs.10 agosto 2018, n. 101;
  - ✓ dalla Delibera del Garante per le "Linee guida per i trattamenti di dati personali nell'ambito delle sperimentazioni cliniche di medicinali" del 24 luglio 2008 e successive modifiche,

tratterà i dati personali per perseguire l'obiettivo dello studio.

**TITOLARE DEL TRATTAMENTO** *(art. 13, par. 1, lett. a del GDPR)*
I dati del Promotore sono i seguenti:

- Largo Francesco Vito, n. 1 – 00168 – Roma
- Tel 06 30151
- PEC: protocollo.generale.gemelli@pec.it

**RESPONSABILE DELLA PROTEZIONE DEI DATI (RPD O DPO)** *(art. 13, par. 1, lett. b del GDPR)*
È possibile rivolgersi al responsabile della protezione dei dati, designato dal titolare del trattamento ai sensi dell'art. 37 del GDPR, è contattabile scrivendo alla sua attenzione presso la sede del titolare ed anche scrivendo all'indirizzo di posta elettronica: dpo@policlinicogemelli.it e all'indirizzo PEC: dpo.gemelli@pec.it. Tuttavia, si precisa che il Promotore tratterà soltanto i dati pseudonimizzati dei soggetti arruolati.

**BASE GIURIDICA E FINALITÀ DEL TRATTAMENTO** *(art. 13, par. 1, lett. c del GDPR)*
I dati *personali* (qualsiasi informazione relativa alla persona fisica che la rende identificata o identificabile), quelli appartenenti a categorie particolari (art. 9 del GDPR) relativi allo *stato di salute* saranno trattati previo rilascio del consenso quale presupposto di liceità del trattamento ai sensi degli artt. 6, par. 1, lett. a) e l'art. 9, par. 2 lett. a) del GDPR. In particolare, i predetti dati, saranno trattati per le seguenti finalità:

☐ ricerca medica anche con sperimentazione clinica di medicinali effettuata sulla base di un progetto, oggetto di motivato parere favorevole del competente Comitato Etico **(studio prospettivo/interventistico);**

☐ conduzione di uno studio effettuato con dati raccolti in precedenza a fini di cura della salute o per l'esecuzione di precedenti progetti di ricerca, ovvero progetti ricavati da campioni biologici prelevati in precedenza per finalità di tutela della salute, oggetto di motivato parere favorevole del competente Comitato Etico **(studio retrospettivo/osservazionale);**

■ ricerca scientifica e statistica finalizzata alla tutela della salute della collettività in campo medico, biomedico ed epidemiologico, survey **(osservazionale prospettico);**

☐ la costituzione, integrazione e/o mantenimento, di un Registro, secondo il titolo dello studio indicato a pag. 1.

**CONFERIMENTO DEI DATI** *(art. 13, par. 1, lett. e del GDPR)*
La partecipazione allo Studio avviene su base volontaria previo rilascio del consenso, pertanto, il mancato consenso al trattamento dei dati ai fini della partecipazione al progetto non consentirà l'arruolamento.
I dati potranno essere comunicati tra i soggetti che agiscono quali Titolari autonomi del trattamento nell'ambito dello Studio per le finalità sopra indicate, nella misura in cui rivestano il ruolo di centro partecipante e le operazioni di comunicazione dei dati siano indispensabili per la conduzione dello Studio stesso. I dati personali non saranno diffusi, se non in forma anonima e/o aggregata in modo da non essere più riconducibili ad alcun interessato.

Come previsto dalla normativa sulle sperimentazioni cliniche, i Suoi dati potranno inoltre essere trasmessi al Comitato Etico e alle competenti autorità, con modalità tali da garantirne la riservatezza.

Qualora risulti necessario ai fini dello studio, i dati potranno essere trasmessi in paesi terzi non appartenenti all'Unione Europea. In tal caso, il trasferimento dei dati avverrà nel rispetto delle norme di cui al Capo V del GDPR (art. 44 e seguenti), in modo tale da garantire un adeguato livello di tutela dei dati personali stabilito in fase contrattuale anche mediante specifiche clausole.

Con Suo previo consenso, i dati che emergeranno dai risultati delle analisi, qualora comportino un beneficio concreto e diretto in termini di terapia, prevenzione o di consapevolezza delle Sue scelte future, potranno essere resi noti a Lei o anche alle persone da Lei espressamente indicate nel presente modulo.

**RICERCA FUTURA** *(art 5 par. 1 lett. b) del GDPR)*
Conformemente all'art. 5 par. 1, lett. b) e al Considerando 33 del GDPR, gli interessati possono esprimere uno specifico consenso ad un ulteriore utilizzo dei dati personali per future attività di ricerca, riguardanti la medesima patologia oggetto dello studio, in quanto attività compatibili con le finalità per le quali i dati sono stati raccolti.
La manifestazione del consenso alla ricerca futura è libera e pertanto il diniego non pregiudicherà in alcun modo la partecipazione al presente studio né le cure prestate.

**MODALITÀ DI TRATTAMENTO DEI DATI** *(art. 13, par. 1, lett. f del GDPR)*
Il trattamento dei dati prevede lo svolgimento di una serie di operazioni (raccolta, registrazione, conservazione, consultazione e modificazione dei dati personali, etc.) mediante strumenti manuali ed informatici con logiche strettamente correlate alle finalità per le quali i dati sono stati raccolti, e comunque, in modo da garantire in ogni fase del trattamento la sicurezza, la riservatezza, l'integrità e la disponibilità dei dati stessi.
Fermo restando l'adozione di misure tecniche e organizzative atte a garantire un livello di sicurezza adeguato al rischio, sono attuate specifiche misure e accorgimenti tecnici al fine di incrementare il livello di sicurezza dei dati trattati per l'esecuzione dello studio. L'accesso ai dati sarà effettuato mediante idonei sistemi di autenticazione e autorizzazione, per il personale preposto al trattamento in funzione dei ruoli ricoperti e delle esigenze di accesso e trattamento. Inoltre, saranno adottate tecniche di pseudonimizzazione e altre soluzioni tali da non rendere direttamente riconducibili i dati dell'interessato.

I dati raccolti dal Centro di Sperimentazione saranno contraddistinti da un codice alfanumerico che sostituirà il nominativo del soggetto interessato, cosicché soltanto il medico ed i soggetti autorizzati potranno associare questo codice al nominativo del partecipante allo studio (psudonimizzazione).

Anche i campioni biologici saranno contrassegnati con un codice alfanumerico che garantirà coerenza e consistenza dei dati. Le informazioni relative ai campioni saranno trattati nel rispetto delle norme

etico–giuridiche e con i più elevati standard tecnologici, garantendo elevati livelli di sicurezza nel trattamento dei dati personali dei partecipanti allo Studio .

I dati saranno trattati esclusivamente da personale a tal fine autorizzato e soggetto al segreto professionale e all'obbligo legale di riservatezza nel rispetto della tutela dei diritti e della dignità dell'interessato.
Nell'ambito dello Studio, i Suoi dati potranno essere trattati con la collaborazione di soggetti nominati "Responsabili del Trattamento" (soggetti esterni che trattano dati per conto del Titolare) nel rispetto delle disposizioni dell'art. 28 del GDPR.

I Suoi dati personali non verranno utilizzati per scopi di profilazione o per prendere decisioni automatizzate che possano comportare un rischio significativo per Lei.

**CONSERVAZIONE** *(art. 13, par. 2, lett. a del GDPR)*
I dati forniti saranno conservati per un arco di tempo non superiore a quello necessario per conseguire le finalità per le quali sono stati trattati, e comunque per 7 anni ai sensi dell'art. 58 del Regolamento UE n. 536/2014 termine dello studio.
Al termine di tale periodo i predetti dati saranno cancellati e i campioni saranno distrutti.

**ESERCIZIO DEI DIRITTI** *(art. 13, par. 2, lett. b del GDPR)*
Ai sensi degli artt. 15, 16, 17, 18, e 21 del GDPR EU 2016/679, l'interessato:

a) ha il diritto di chiedere al Titolare del trattamento l'accesso ai dati personali, la rettifica, l'integrazione, la cancellazione degli stessi, la limitazione del trattamento dei dati che lo riguardano o di opporsi al trattamento degli stessi qualora ricorrano i presupposti previsti dal GDPR;

b) ha il diritto di proporre un reclamo al Garante per la protezione dei dati personali, seguendo le procedure e le indicazioni pubblicate sul sito web ufficiale dell'Autorità: www.garanteprivacy.it.

L'interessato potrà in qualsiasi momento esercitare i diritti di cui sopra contattando il titolare ed il suo responsabile della protezione dei dati ai recapiti riportati nella presente informativa.

Il Titolare s'impegna a comunicare al soggetto interessato eventuali variazioni che si dovessero rendere necessarie, nelle operazioni di trattamento dei dati personali effettuate nell'ambito delle finalità sopra descritte. L'interessato potrà in ogni momento e senza fornire alcuna giustificazione, revocare il consenso e interrompere la Sua partecipazione allo studio; in tal caso non saranno più raccolti ulteriori dati che la riguardano, ferma restando l'utilizzazione di quelli eventualmente già raccolti per determinare, senza alterarli, i risultati della ricerca. La revoca del consenso non pregiudica la liceità del trattamento basata sul consenso prestato prima della revoca.
In caso di interruzione del trattamento, i campioni biologici eventualmente prelevati ai fini dello studio saranno distrutti.

**DIRITTO DI PROPORRE RECLAMO ALL'AUTORITÀ DI CONTROLLO** *(art. 13, par. 2, lett. d del GDPR)*
Ai sensi dell'art. 77 del GDPR e fatto salvo ogni altro ricorso amministrativo o giurisdizionale, ove l'interessato ritenga che il trattamento che lo riguardi violi lo stesso Regolamento, ha il diritto di proporre reclamo all'autorità di controllo competente, individuata nel Garante per la protezione dei dati personali ai sensi dell'art. 153 del D.Lgs. 196/03 per come novellato dal D.Lgs. 101/18, secondo le modalità descritte sul sito web istituzionale www.gpdp.it.

**Consenso al Trattamento dei dati per Finalità di Ricerca Scientifica per Adulti**
*Ai sensi dell'art. 7 del GDPR*

Preso atto dell'informativa resa ai sensi dell'art. 13 del GDPR, di cui il presente modulo costituisce parte integrante

Il/la sottoscritto/a Nome____________________________Cognome __________________________

Codice Fiscale ___________________________________, tel. ________________________________

In qualità di Interessato

□ dà il proprio consenso □ nega il proprio consenso
al trattamento dei dati per finalità relative alla Ricerca, come indicate nell'informativa.

□ dà il proprio consenso □ nega il proprio consenso
al trattamento dei dati per finalità di ricerca futura nei limiti indicati nell'informativa.

□ dà il proprio consenso □ nega il proprio consenso
affinché i risultati delle analisi e di eventuali scoperte inattese che emergano durante le attività di sperimentazione siano comunicate a:

□ me medesimo

□ familiare (Cognome e nome _____________________________) Contatto: ___________________

□ convivente /coniuge (Cognome e nome ___________________) Contatto: ___________________

□ medico di famiglia (Cognome e nome ___________________ ) Contatto: ___________________

Firma dell'Interessato_____________________Data______________________

STROBE Statement—Checklist of items that should be included in reports of ***cohort studies***

| | Item No | Recommendation |
|---|---|---|
| **Title and abstract** | p. 1,2 | (*a*) Indicate the study's design with a commonly used term in the title or the abstract |
| | | (*b*) Provide in the abstract an informative and balanced summary of what was done and what was found |
| **Introduction** | | |
| Background/rationale | p. 2-4 | Explain the scientific background and rationale for the investigation being reported |
| Objectives | p. 4-5 | State specific objectives, including any prespecified hypotheses |
| **Methods** | | |
| Study design | p. 5 | Present key elements of study design early in the paper |
| Setting | p. 5,6 | Describe the setting, locations, and relevant dates, including periods of recruitment, exposure, follow-up, and data collection |
| Participants | p. 6 | (*a*) Give the eligibility criteria, and the sources and methods of selection of participants. Describe methods of follow-up |
| | | (*b*) For matched studies, give matching criteria and number of exposed and unexposed |
| Variables | p. 6,7 | Clearly define all outcomes, exposures, predictors, potential confounders, and effect modifiers. Give diagnostic criteria, if applicable |
| Data sources/ measurement | p. 6,7 | For each variable of interest, give sources of data and details of methods of assessment (measurement). Describe comparability of assessment methods if there is more than one group |
| Bias | p. 7 | Describe any efforts to address potential sources of bias |
| Study size | p. 7 | Explain how the study size was arrived at |
| Quantitative variables | p. 6,7 | Explain how quantitative variables were handled in the analyses. If applicable, describe which groupings were chosen and why |
| Statistical methods | p. 7 | (*a*) Describe all statistical methods, including those used to control for confounding |
| | | (*b*) Describe any methods used to examine subgroups and interactions |
| | | (*c*) Explain how missing data were addressed |
| | | (*d*) If applicable, explain how loss to follow-up was addressed |
| | | (*e*) Describe any sensitivity analyses |
| **Results** | | |
| Participants | p. 7,8 | (a) Report numbers of individuals at each stage of study—eg numbers potentially eligible, examined for eligibility, confirmed eligible, included in the study, completing follow-up, and analysed |
| | | (b) Give reasons for non-participation at each stage |
| | | (c) Consider use of a flow diagram |
| Descriptive data | p. 7-15 | (a) Give characteristics of study participants (eg demographic, clinical, social) and information on exposures and potential confounders |
| | | (b) Indicate number of participants with missing data for each variable of interest |
| | | (c) Summarise follow-up time (eg, average and total amount) |
| Outcome data | p. 7-15 | Report numbers of outcome events or summary measures over time |
| Main results | p. 7-15 | (*a*) Give unadjusted estimates and, if applicable, confounder-adjusted estimates and their precision (eg, 95% confidence interval). Make clear which confounders were adjusted for and why they were included |
| | | (*b*) Report category boundaries when continuous variables were categorized |
| | | (*c*) If relevant, consider translating estimates of relative risk into absolute risk for a |

| | | |
|---|---|---|
| | | meaningful time period |
| Other analyses | NA | Report other analyses done—eg analyses of subgroups and interactions, and sensitivity analyses |
| **Discussion** | | |
| Key results | p. 15 | Summarise key results with reference to study objectives |
| Limitations | p. 16 | Discuss limitations of the study, taking into account sources of potential bias or imprecision. Discuss both direction and magnitude of any potential bias |
| Interpretation | p. 16 | Give a cautious overall interpretation of results considering objectives, limitations, multiplicity of analyses, results from similar studies, and other relevant evidence |
| Generalisability | p. 16,17 | Discuss the generalisability (external validity) of the study results |
| **Other information** | | |
| Funding | p. 17 | Give the source of funding and the role of the funders for the present study and, if applicable, for the original study on which the present article is based |

*Give information separately for exposed and unexposed groups.

**Note:** An Explanation and Elaboration article discusses each checklist item and gives methodological background and published examples of transparent reporting. The STROBE checklist is best used in conjunction with this article (freely available on the Web sites of PLoS Medicine at http://www.plosmedicine.org/, Annals of Internal Medicine at http://www.annals.org/, and Epidemiology at http://www.epidem.com/). Information on the STROBE Initiative is available at http://www.strobe-statement.org.
